# The Upregulation of Genomic Imprinted DLK1-Dio3 miRNAs in Murine Lupus Is Associated with Global DNA Hypomethylation

**DOI:** 10.1371/journal.pone.0153509

**Published:** 2016-04-12

**Authors:** Rujuan Dai, Ran Lu, S. Ansar Ahmed

**Affiliations:** Infectious Disease Research Facility (IDRF), Department of Biomedical Sciences and Pathobiology, Virginia-Maryland College of Veterinary Medicine (VMCVM), Virginia Tech, Blacksburg, Virginia, United States of America; INSERM-Université Paris-Sud, FRANCE

## Abstract

Epigenetic factors such as DNA methylation and microRNAs (miRNAs) are now increasingly recognized as vital contributors to lupus etiology. In this study, we investigated the potential interaction of these two epigenetic factors in lupus-prone MRL-*lpr* mice. We recently reported dysregulated expression of miRNAs in splenocytes of MRL-*lpr* mice. Here, we report that a majority of the upregulated miRNAs in MRL-*lpr mice* is located at the genomic imprinted DLK1-Dio3 domain. Further, we show a differential magnitude of upregulation of DLK1-Dio3 miRNA cluster in purified splenic CD4^+^ T, CD19^+^ B, and splenic CD4^-^CD19^-^ cells from MRL-*lpr* lupus mice when compared to control MRL mice. MRL-*lpr* splenocytes (especially CD19^+^ and CD4^-^CD19^-^ subsets) were hypomethylated compared to cells from control, MRL mice. We further show that deliberate demethylation of splenocytes from control MRL mice, but not from MRL-*lpr* lupus mice, with specific DNA methylation inhibitor 5-Aza-2’-deoxycytidine significantly augmented DLK1-Dio3 miRNAs expression. These findings strongly indicate that the upregulation of DLK1-Dio3 miRNAs in lupus splenic cell subsets is associated with reduced global DNA methylation levels in lupus cells. There was a differential upregulation of DLK-Dio3 miRNAs among various demethylated splenic cell subsets, which implies varied sensitivity of DLK1-Dio3 miRNA cluster in these cell subsets to DNA hypomethylation. Finally, inhibition of select DLK1-Dio3 miRNA such as miR-154, miR-379 and miR-300 with specific antagomirs significantly reduced the production of lupus-relevant IFNγ, IL-1β, IL-6, and IL-10 in lipopolysaccharide (LPS) activated splenocytes from MRL-*lpr* mice. Our study is the first to show that DNA methylation regulates genomic imprinted DLK1-Dio3 miRNAs in autoimmune lupus, which suggests a connection of DNA methylation, miRNA and genomic imprinting in lupus pathogenesis.

## Introduction

Systemic lupus erythematosus (SLE) is a female predominant autoimmune disease that is characterized by the production of autoantibodies against various nuclear antigens and multi-organ damage. While extensive studies from past decades have unraveled many lupus predisposed genes in both human and murine lupus, the induction of SLE cannot be solely attributed to genetic defects [1–3]. Therefore, much attention has been shifted recently to understand the important contribution of epigenetics to lupus etiology. Epigenetics is the study of heritable changes of gene expression and physiological traits that are not caused by DNA sequence changes [4]. It is now well acknowledged that in addition to genetic factors, different epigenetic factors such as histone modification, DNA methylation, and miRNAs are also critically involved in SLE pathogenesis [5, 6].

DNA methylation is a biochemical process that adds a methyl group to 5’ cytosine within a CpG dinucleotide context. Being the most extensively studied epigenetic mechanism so far, DNA methylation regulates gene expression at the transcriptional level and is critically involved in the regulation of many key biological processes including embryonic development, genome expression, X-chromosome inactivation (XCI), genomic imprinting, and chromosome stability [7]. Abnormal DNA methylation level is linked with a growing number of human diseases, which include cancers, genetic imprinting disorders, and also autoimmune diseases. Reduced expression of DNA (cytosine-5)-methyltransferase (DNMT)s and global DNA hypomethylation are observed in both human and murine lupus CD4^+^ T cells, which are associated with increased expression of autoimmune associated genes such as *CD40 ligand* (CD40L) and *TNFSF7* (CD70) in lupus T cells [8–10]. The importance of DNA hypomethylation in lupus was supported by the findings that demethylation of normal human and murine CD4^+^ T cells with a specific DNA methylation inhibitor induced auto-reactivity in these cells, and deliberate adoptive transfer of demethylated CD4^+^ T cells into syngeneic recipient mice induced lupus-like disease [11]. The recent genome-wide DNA methylation profiling studies revealed a persistent hypomethylation of Type I interferon-related genes in CD4^+^ T cells, suggesting an involvement of epigenetic mechanisms in heightened type I interferon signaling and sensitivity in lupus T cells [12, 13]. Further, the discordance of lupus incidence in monozygotic twins is also associated with the changes of DNA methylation pattern for numerous genes [14]. Together, it is evident that DNA methylation plays a critical role in lupus pathogenesis.

Another epigenetic factor that has been extensively investigated recently is a group of small non-coding RNAs called microRNAs (miRNAs) that demonstrate notable regulatory role in genome expression. It is thus not surprising that miRNAs are now regarded as key regulators of immune system development and function. Disruption of miRNA expression or function could cause immune tolerance breakdown and consequently lead to the development of autoimmunity [15–18]. The dysregulated miRNA expression has been identified in both human and murine lupus, and the important pathogenic contribution of dysregulated miRNAs to lupus has been extensively reviewed [19–23]. The interaction between DNA methylation and miRNA regulation in lupus is observed in recent studies. Increased miR-21, miR-148a, and miR-126 in lupus CD4^+^ T cells reduced the expression of DNMT1 directly or indirectly, leading to DNA hypomethylation and overexpression of autoimmune-associated methylation-sensitive genes such as CD70, lymphocyte function-associated antigen 1 (LFA-1), and CD11a [24–26]. On the other hand, abnormal DNA methylation levels could also cause miRNA dysregulation in autoimmune lupus. The overexpression of X-chromosome linked miRNAs in T cells from women with active lupus is linked with demethylation of inactivated X-chromosome, suggesting an involvement of X-chromosome demethylation in female predominance of lupus [27].

In our previous study of profiling dysregulated miRNAs in different murine lupus models with miRNA microarray, we found that 11 out of the 17 upregulated miRNAs in splenocytes of MRL-*lpr* mice belong to the largest miRNA cluster located at the genomic imprinted DLK1-Dio3 region [28]. The highly conserved mammalian DLK1-Dio3 region spans over 800 kb on mouse chromosome 12F1 and human chromosome 14q32, and is defined by paternally expressed protein-coding gene Delta-like homologue 1 (*Dlk1*) and type III iodothyronine deiodinase (*Dio3*) [29, 30]. Interstingly, the DLK1-Dio3 domain contains several non-protein coding gene clusters that are exclusively expressed from maternally inherited chromosome [29–31]. These include Gene-trap line 2 (*Gtl2*, human ortholog *MEG3*: maternally expressed gene 3), RNA imprinted and accumulated in nucleus (*RIAN*, human ortholog *MEG8*), antisense retrotransposon-like gene 1 (*asRTL1*), and microRNA-containing gene (*Mirg*). So far, 54 miRNAs have been identified in human DLK1-Dio3 domain, of which most are mapped in *asRTL1* and *Mirg* region. The epigenetic silencing of the imprinted DLK1-Dio3 miRNA cluster has been documented in melanoma, ovarian, and bladder cancer, and many DLK1-Dio3 miRNAs have tumor suppressor function [32–34]. Nevertheless, there is thus far limited understanding of the regulation and immune regulatory function of DLK1-Dio3 miRNAs in lupus.

In this study, we reported the differential upregulation of DLK1-Dio3 miRNAs in purified splenic CD4^+^ T, CD19^+^ B, and CD4^-^CD19^-^ cells from MRL-*lpr* mice when compared to control MRL mice. We further demonstrated that the expression of DLK1-Dio3 miRNAs in immune cells is subjected to DNA methylation regulation and that the upregulation of DLK1-Dio3 miRNAs in MRL-*lpr* splenic cells is associated with global DNA hypomethylation. Although the target gene/pathways remains to be experimentally determined, we demonstrated here that inhibition of specific DLK1-miRNAs with antagomirs reduced the production of lupus-relevant cytokines in LPS-activated splenocytes from MRL-*lpr* mice. This indicates a potential role of genomic imprinted DLK1-Dio3 miRNAs in regulation of inflammation in lupus. Together, our novel data suggests interaction between two critical epigenetic pathways, DNA methylation and miRNA regulation, in lupus pathogenesis.

## Materials and Methods

### Ethics statement and mice

All animal experimental procedures and housing have been approved by the Institutional Animal Care and Use Committee (IACUC) of Virginia Tech (Protocol ID# 13-122-CVM). Genetically lupus-prone MRL/MpJ-Fas^lpr^/J (MRL-*lpr*, stock# 000485) and control MRL/MpJ (MRL, stock# 000486) breeders were purchased from The Jackson Laboratory, ME, USA and bred in house. Only female MRL and MRL-*lpr* mice were used in this study. The experimental mice were euthanized by cervical dislocation in strict accordance with approved IACUC protocol and regulation. To minimize suffering and to ensure a successful euthanasia of mice within seconds, cervical dislocation was carried out only by well-trained and approved research staffs. All mice were housed in our AAALAC certified animal facility at the Virginia-Maryland College of Veterinary Medicine (VMCVM), Virginia Tech. Mice were fed with a commercial 7013 NIH-31 Modified 6% Mouse/Rat Sterilizable Diet (Harlan Laboratory, Madison, WI, USA) and gave water *ad libitum*.

### Splenocyte preparation, splenic CD4^+^ T and CD19^+^ B cell purification

Splenocytes were isolated using standard procedures described in detail previously [35, 36]. Per the manufacturer’s instruction, splenic CD4^+^ T cells and CD19^+^ B cells were purified from freshly isolated splenocytes sequentially by positive selection with anti-CD4 (L3T4) and anti-CD19 MicroBeads (Miltenyi Biotec, San Diego, CA, USA) respectively. Briefly, the splenocytes were firstly stained with MicroBeads conjugated with anti-mouse CD4 antibodies to separate CD4^+^ T cells. The negative effluent fractions (CD4^-^ cells) were collected and stained with MicroBeads conjugated with anti-CD19 antibodies to isolate CD19^+^ B cells. The double negative effluent fractions that contain CD4^-^CD19^-^ splenic cells were also collected for analysis. The purity of isolated splenic CD4^+^ T cells (90–98%) and CD19^+^ B cells (80–95%) was confirmed by flow cytometry after staining the isolated cells with ef450 conjugated anti-CD4 and APC conjugated anti-CD19 antibodies (eBioscience, San Diego, CA, USA), respectively. The splenocytes and purified CD4^+^ T cells were adjusted to 5x10^6^/ml and 2.5x10^6^/ml respectively with complete RPMI medium (Mediatech Inc, Manassas, VA, USA) containing 10% charcoal-stripped fetal bovine serum (Atlanta Biologicals, Flowery Branch, GA, USA), 2 mM L-glutamine (HyClone Labs Inc, Logan, UT, USA), 100 IU/ml penicillin and 100 μg/ml streptomycin (HyClone), and 1% non-essential amino acids (HyClone) before seeding into cell culture plate for treatment.

### DNA demethylation treatment

To determine the effect of DNA demethylation on the expression of DLK1-Dio3 miRNAs, the splenocytes (2.5–5x10^6^) and purified CD4^+^ T cells (1.25–2.5x10^6^) were cultured with medium containing vehicle solution (dimethyl sulfoxide, DMSO), 2μM or 5μM of DNA demethylating drug 5-aza-2'-deoxycitydine (5-aza-CdR, from Sigma-Aldrich, St. Louis, MO, US) for 72hrs (medium group). Aliquots of cells received Concanavalin A (Con A, 5 μg/ml, from Sigma-Aldrich) stimulation plus 5-aza-CdR treatment at same time for 72hrs (Con A group). To test the effect of 5-aza-CdR treatment on the cell viability, after 72hrs of treatment, aliquots of cells (5x10^5^ in 100 μl buffer) were stained with propidium iodide (PI, 1 μg/ml) and then subjected to Flow cytometric analysis. While 5-aza-CdR treatment has no obvious effect on the viability of inactivated splenocytes, it reduced the cell viability in inactivated CD4^+^ T cells and in both Con A stimulated splenocytes and CD4^+^ T cells (S1 Fig). After treatment, the cell pellets were collected to prepare RNA for analysis of DLK1-Dio3 miRNA expression. In a separate experimental setting, the splenocytes (25–30 x10^6^) were treated with Con A plus 5-aza-CdR (2μM or 5μM) for 72hrs first, and then subjected to CD4^+^ T cell and CD19^+^ B cell purification sequentially and miRNA expression analysis. The effluent fraction containing CD4^-^CD19^-^ splenic cells was also collected for analysis.

### RNA isolation and miRNA quantification

Per the manufacturer’s instruction, total RNA, containing small RNA, was isolated from splenocytes and purified splenic cell subsets with miRNeasy isolation kit (Qiagen, Valencia, CA, USA). On-column DNAase digestion was performed during RNA isolation to remove any contaminated residual DNA. The RNA concentration was measured with NanoDrop 2000 spectrophotometer (Thermo Scientific, Waltham, MA, USA). The miRNA expression level was quantified by using Taqman miRNA assay (Life technologies, Grand Island, NY, USA). The relative miRNA expression level was calculated with formula 2^-^ΔΔCt (Livak) method by normalizing to endogenous small RNA control, snoRNA 202 [37, 38].

### DNA isolation and global DNA methylation analysis

The whole genomic DNA was isolated from splenic cells with DNeasy Blood and Tissue Kit (Qiagen). The DNA concentration was measured with NanoDrop 2000 spectrophotometer. The 5-mC DNA ELISA Kit (ZYMO Research, Irvine, CA, USA) was used to measure the global DNA methylation level. Briefly, 100ng DNA of each sample was brought up to 100 μl volume with 5-mC coating buffer, denatured at 98°C, and then coated into 96-well assay plate. After washing, the coated DNA was incubated with an antibody mix consisting of anti-5-Methylcytosine antibody and secondary antibody. After antibody incubation, the plate was washed, and HRP developer solution was added to develop color signal. The absorbance was measured by reading the plate at 405nm on a SpectraMax M5 Microplate reader (Molecular Devices, Sunnyvale, CA, USA). The percentage of 5-mC in each DNA sample was quantified with a standard curve that was generated with kit-provided positive control (100% methylation) and negative control (0% methylation).

### Antagomir treatment

Antagomirs are chemically engineered single strand RNA oligonucleotides that silence specific miRNA *in vitro* in cultured cells and *in vivo* in animals efficiently [39, 40]. As previously described [39], antagomirs against specific DLK1-Dio3 miRNAs were designed based on mature murine miRNA sequence from miRBase (http://www.mirbase.org/), and then synthesized by GE Dharmacon (Lafayette, CO, USA). The sequences of scrambled control specific DLK1-Dio3 miRNAs were listed in S1 Table.

As previously reported [40], to inhibit function of a specific miRNA in splenocytes, freshly isolated splenocytes were washed with PBS with 0.5% BSA, resuspended in serum free Accell siRNA delivery medium (GE Dharmacon) supplemented with 1μM specific antagomir or scrambled control antagomir at 10x10^6^/ml, and then incubated in the cell incubator (37°C, 5% CO2) for 1.5–2 hrs. After incubation, the treated cells were pelleted and resuspended with complete RPMI medium supplemented with 0.1μM respective antagomirs at 5x10^6^/ml and plated into 24 well plate for culture. Twenty-four hours after antagomirs treatment, the cells were stimulated with lipopolysaccharide (LPS, 500ng/ml, from Sigma-Aldrich) for the designated time. The supernatant were collected for analysis of cytokine production.

### Multiplex Cytokine Assay

Ciraplex® Chemiluminescent Assay kit (Aushon Biosystem, Billerica,MA, USA) was used to simultaneously quantify the levels of multiple cytokines including IFNγ, IL-1β, IL-6, IL-10, and TNFα in cell culture supernatants per the manufacturer’s instructions. The image of chemiluminescent array plate was captured with Cirascan image system (Aushon) and the image data was processed with Cirasoft software.

### Statistical Analysis

All values in the graphs were given as means ± SEM. Two tailed, unpaired *t* tests were performed to assess statistical significance of DLK1-Dio3 mRNA expression in splenic cells between MRL and MRL-*lpr* mice. Paired student *t* tests were used to assess statistical significance of 5-aza-CdR treatment on miRNA expression in splenic cells (vehicle *vs* 5-aza-CdR).

## Results

### Genomic imprinted DLK1-Dio3 miRNAs are markedly upregulated in lupus-prone MRL-*lpr* mice

**Fig 1:**
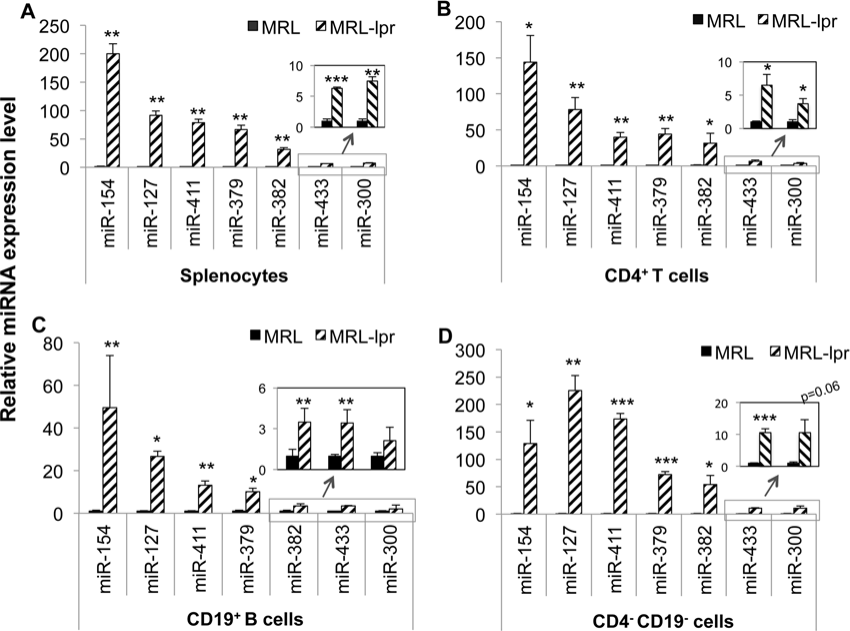
DLK1-Dio3 miRNAs are highly upregulated in splenic cells from MRL-*lpr* lupus mice when compared to control MRL mice. The miRNA expression in splenocytes (A), purified splenic CD4^+^ T cells (B), CD19^+^ B cells (C), and double negative effluent fraction splenic CD4^-^CD19^-^ cells (D) from MRL and MRL-*lpr* mice (13–16 wks old) were quantified by Taqman miRNA assays. The graphs show mean ± SEM (n = 3 each). Unpaired student *t* tests (MRL *vs* MRL-*lpr*) were preformed; *, *p* < 0.05; **, *p* < 0.01; and ***, *p* < 0.001.

By using a miRNA microarray profiling assay, we have previously reported that 49 miRNAs were dysregulated (17 upregulated and 32 downregulated) in splenocytes from MRL-*lpr* mice when compared to MRL control mice [28]. Impressively, of the 17 upregulated miRNAs in MRL-*lpr* mice, 11 miRNAs (miR-154, miR-127, miR-379, miR-382, miR-433, miR-300, miR-376b, miR-394, miR-299, miR-495, and miR-329) are located at a genomic imprinted DLK1-Dio3 region. In this study, we performed Taqman miRNA assays to confirm the upregulation of selected DLK1-Dio3 miRNAs such as miR-154, miR-127, miR-379, miR-382, miR-300, and miR-433 in MRL-*lpr* splenocytes. We also demonstrated that an additional DLK-Dio3 miRNA, miR-411, which was not identified by previous miRNA microarray profiling assay, was also markedly increased in MRL-*lpr* splenocytes (Fig 1A). This suggests the possibility of upregulation of the entire DLK1-Dio3 miRNA cluster in MRL-*lpr* splenocytes. Further investigation of the expression of whole DLK1-Dio3 miRNA cluster in MRL and MRL-*lpr* splenocytes is necessary to confirm this view. Considering the cell-specific expression and function of miRNA, we further investigated the expression of aforementioned DLK1-Dio3 miRNAs in various purified splenic cell subsets. As indicated, the expression levels of these miRNAs were significantly upregulated in purified splenic CD4^+^ T cells (Fig 1B), CD19^+^ B cells (Fig 1C), and CD4^-^CD19^-^ cells (splenic cells after depletion of CD4^+^ T and CD19^+^B cells, Fig 1D). By comparing the expression level of a specific DLK-Dio3 miRNA across different splenic immune cell subsets, we found that all the examined DLK1-Dio3 miRNAs displayed the lowest expression level in splenic CD19^+^ B cells in both MRL and MRL-*lpr* mice (S2A and S2B Fig). Correspondingly, the magnitude of upregulation of DLK1-Dio3 miRNA in purified CD19^+^ B cells is much smaller when compared to either CD4^+^ T cells or CD4^-^CD19^-^ cells. Taken together, our data demonstrated a substantial upregulation of genomic imprinted DLK1-Dio3 miRNAs in splenic cells from MRL-*lpr* lupus mice.

### Splenic cells from MRL-*lpr* mice have reduced global DNA methylation levels

**Fig 2:**
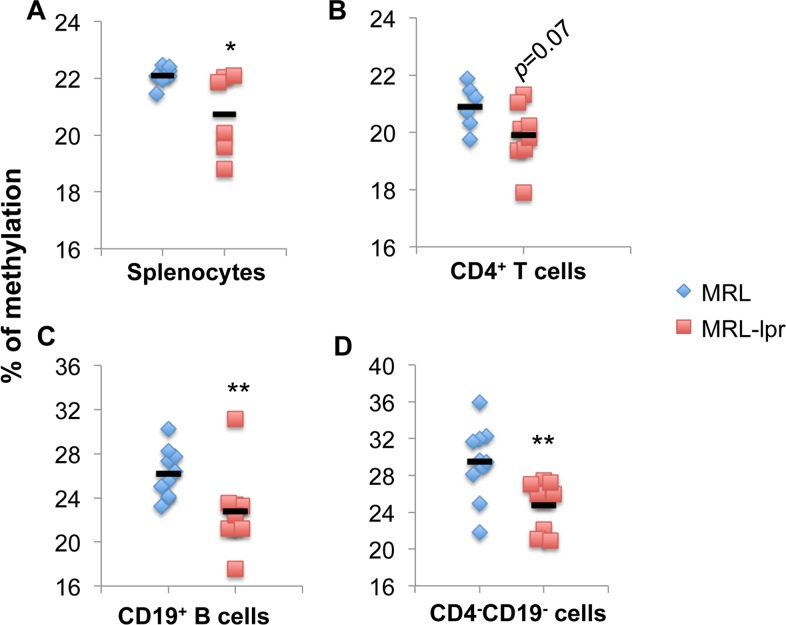
The global DNA methylation levels are reduced in splenic cells from MRL-*lpr* lupus mice. The DNA methylation levels in splenocytes (A), purified splenic CD4^+^ T cells (B), CD19^+^ B cells (C), and negative effluent fraction CD4^-^CD19^-^ cells (D) from MRL and MRL-*lpr* mice (13–16 wks old) were measured with the 5-mc DNA ELISA kit. The graphs present the percentage of methylation of each sample (n≥6). The mean DNA methylation value in each sample group was indicated by black bar. Unpaired student *t* tests (MRL vs MRL-*lpr*) were preformed; *, p < 0.05; and **, p < 0.01.

To understand whether altered DNA methylation contributes to the upregulation of genomic imprinted DLK1-Dio3 miRNAs in lupus splenic cells, we measured the global DNA methylation levels in splenocytes, purified splenic CD4^+^ T cell, CD19^+^ B cells, and splenic CD4^-^CD19^-^ cells from MRL and MRL-*lpr* mice. Compared to control MRL mice, MRL-*lpr* splenocytes demonstrated a decreased DNA methylation level (Fig 2A). Consistent with a previous report [9], MRL-*lpr* CD4^+^ T cells have a lower global DNA methylation level than MRL CD4^+^ T cells (Fig 2B), but the difference was not statistically significant (*p = 0*.*07*, Fig 2B). Further, in this study we demonstrated that DNA methylation levels in splenic CD19^+^ B (Fig 2C) cells, and CD4^-^CD19^-^ cells (Fig 2D) were significantly decreased in MRL-*lpr* mice when compared to control MRL mice. These data suggested a potential association between decreased DNA methylation levels with increased DLK1-Dio3 miRNAs expression in lupus.

### DNA demethylation treatment promotes DLK1-Dio3 miRNA expression in splenic cells from MRL mice, but not MRL-*lpr* mice

**Fig 3:**
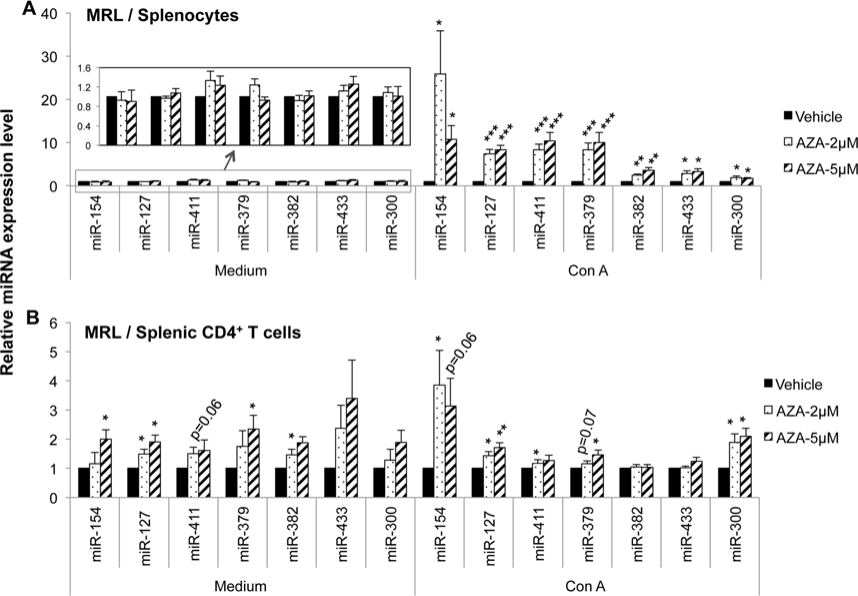
DNA demethylation treatment with 5-aza-CdR significantly increases DLK1-Dio3 miRNAs in splenic cells from MRL mice. The splenocytes (A) and purified CD4^+^ T cells (B) from MRL mice (13–16 wks old) were treated with vehicle solution (DMSO) or 5-aza-CdR (AZA, 2μM or 5μM), with (Con A) or without (medium) Con A (5μg/ml) activation for 72 hrs. The expression of DLK1-Dio3 miRNAs was quantified by Taqman miRNA assays. The graphs show mean ± SEM (n≥4). Paired student *t* tests were performed (Vehicle *vs* AZA); *, *p* < 0.05; **, *p* < 0.01; and ***, *p* < 0.001.

**Fig 4:**
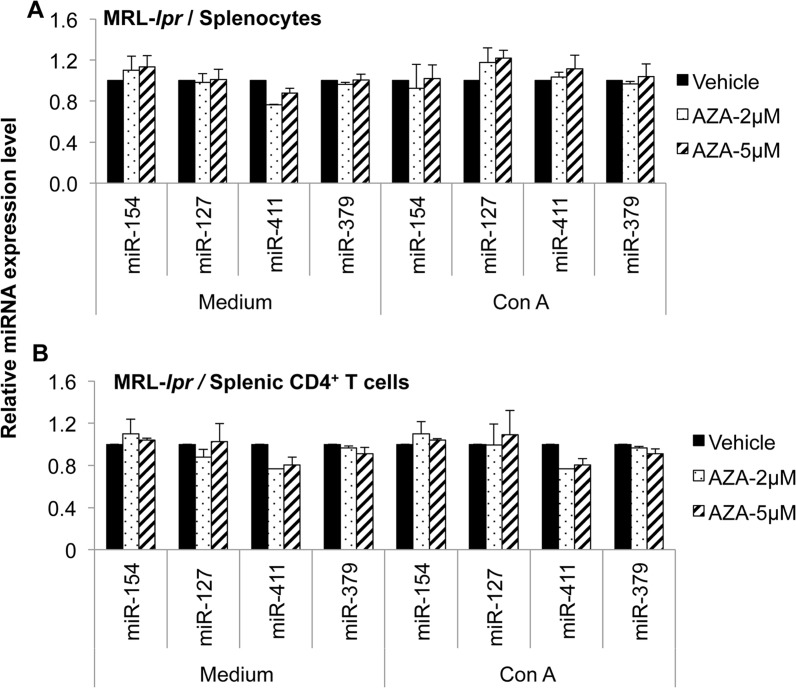
5-aza-CdR treatment has no obvious effect on the expression of DLK1-Dio3 miRNAs in splenic cells from MRL-*lpr* lupus mice. The splenocytes (A) and purified CD4^+^ T cells (B) from MRL-*lpr* mice (13–15 wks old) were treated with 5-aza-CdR and miRNAs were quantified as we described for MRL mice in Fig 3. The graphs show mean ± SEM (n≥ 2).

To further determine whether DLK1-Dio3 miRNAs are subjected to DNA methylation regulation directly, we treated splenocytes and purified CD4^+^ T cell from MRL mice with vehicle, 2 μM or 5μM demethylation drug 5-aza-CdR, in the presence or absence of a lymphocyte mitogen (Con A) for 72 hours, and then quantified the miRNA expression. While DNA demethylation treatment did not induce any DLK1-Dio3 miRNA expression in unstimulated splenocytes (medium, Fig 3A), it did induce the expression of DLK1-Dio3 miRNAs including miR-154, miR-127, miR-379, miR-382, miR-433, and miR-300 substantially in Con A activated splenocytes (Con A, Fig 3A). There was a greater than 10 fold increase for selected DLK1-Dio3 miRNAs such as miR-154, miR-127, miR-411, miR-379 in 5-aza-CdR plus Con A treated MRL splenocytes. There was no noticeable difference in the expression of DLK-Dio3 miRNAs between 2μM and 5μM 5-aza-CdR treated samples. Unlike that we observed in splenocytes, there was a slight but significant increase of several DLK1-Dio3 miRNAs such as miR-154, miR-127, miR-379, and miR-382 even in the unstimulated CD4^+^ T cells from MRL mice (medium). Surprisingly, there was no further increase of DLK1-Dio3 miRNAs in 5-aza-CdR treated, Con A stimulated CD4^+^ T cells (Fig 3B). The deliberate DNA demethylation treatment of either splenocytes or purified CD4^+^ T cells from MRL-*lpr* mice did not affect DLK1-Dio3 miRNA expression (Fig 4A and 4B). We tested only miR-154, miR-127, miR-411, and miR-379 in cells from MRL-*lpr* mice since these four miRNAs were the most upregulated miRNAs in 5-aza-CdR treated splenocytes from MRL control mice. Overall, our data strongly suggested that DLK1-Dio3 miRNA expression is subjected to DNA methylation regulation directly.

### The magnitude of increase in DLK1-Dio3 miRNA expression following DNA demethylation treatment is varied among different splenic cell subsets

**Fig 5:**
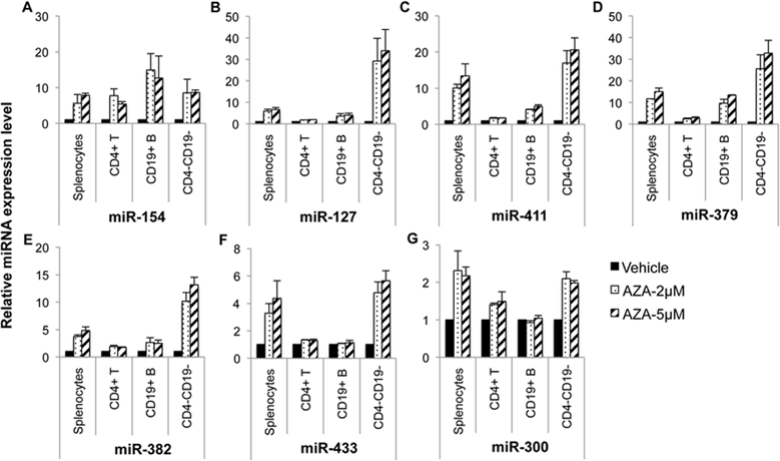
Splenic cell subsets have different sensitivity in response to 5-aza-CdR demethylation treatment to induce DLK1-Dio3 miRNAs. The splenocytes from MRL mice (about 15–16 wks old) were treated with either vehicle solution (vehicle) or 5-aza-CdR (AZA, 2μM or 5μM) plus Con A (5ng/ml). After 72 hrs of treatment, the splenocytes were collected to purify CD4^+^ T, CD19^+^ B cells sequentially. A small aliquot of treated splenocytes was saved as control. The expression levels of miR-154 (A), miR-127 (B), miR-411 (C), miR-379 (D), miR-382 (E), miR-433 (F), and miR-300 (G) in vehicle and 5-aza-CdR treated splenocytes, purified CD4^+^ T cells, CD19^+^ B cells, and splenic CD4^-^CD19^-^ cells were quantified by Taqman miRNA assays. The graphs show means ± SEM (n = 2 each).

The above data indicated that while 5-aza-CdR treatment increased DLK1-Dio3 miRNAs substantially in Con A activated splenocytes, it had a much smaller effect on purified splenic CD4^+^ T cells (Fig 3). This suggested that DLK1-Dio3 miRNA cluster may respond to DNA demethylation treatment differentially in diverse immune cell types. To test this view, we treated splenocytes with 5-aza-CdR plus Con A stimulation for 72 hours first, then purified CD4^+^ T cells and CD19^+^ B cells for miRNA analysis. While miR-154 showed a similar increase in splenocytes and in different splenic immune cell subsets, the other six DLK1-Dio3 miRNAs including miR-127 (Fig 5B), miR-411 (Fig 5C), miR-379 (Fig 5D), miR-382 (Fig 5E), miR-433 (Fig 5F), and miR-300 (Fig 5G) were upregulated more dramatically in CD4^-^CD19^-^ cells when compared to that in purified CD4^+^ T and CD19^+^ B cells. There was no apparent difference of 5-aza-CdR induced DLK1-Dio3 miRNAs expression changes in splenic CD4^+^ T cells between two different approaches: treating purified CD4^+^ T cells directly with 5-aza-CdR (Fig 3B) or purifying CD4^+^ T from demethylated splenocytes (Fig 5) for miRNA expression analysis. These data indicated that the DLK1-Dio3 miRNAs are more sensitive to DNA demethylation treatment in CD4^-^CD19^-^ splenic cells, which were enriched with CD4^-^CD8^+^ lymphocytes and myeloid cells such as macrophage, dendritic cells, and neutrophils.

### Inhibition of selected DLK1-Dio3 miRNAs reduced the production of lupus-related inflammatory cytokines

**Fig 6:**
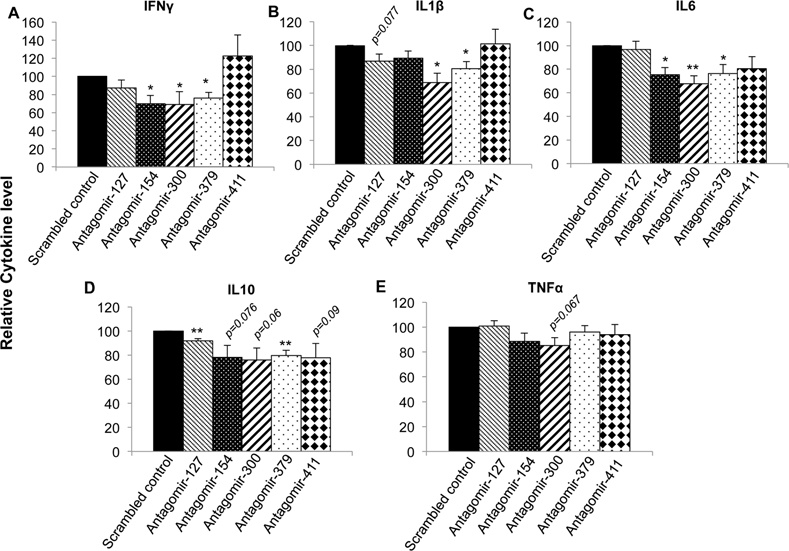
Inhibition of DLK1-Dio3 miRNA significantly reduces lupus-related cytokines in splenocytes from MRL-*lpr* mice. The splenocytes from MRL-*lpr* mice (14–16wks) were treated with either scrambled control antagomirs or specific antagomirs against individual DLK1-Dio3 miRNA for 24hrs, and then stimulated with LPS (500 ng/ml) for 48hrs. The production levels of IFNγ (A), IL1β (B), IL-6 (C), IL-10 (D), and TNFα (E) in the culture supernatants were measured by Ciraplex® Chemiluminescent multiplex cytokine assay. The graphs show means ± SEM (n = 4 each). The cytokine level in specific antagomir-treated cells was shown as the percentage of scrambled control antagomir-treated cells. Paired student *t* tests were performed (scrambled control *vs* specific antagomirs); *, *p* < 0.05; and **, *p* < 0.01.

Abnormal production of inflammatory cytokines such as IFNγ, IL-1β, IL-6, and TNFα is a key characteristic of lupus [41]. We therefore investigated whether DLK1-Dio3 miRNAs play a role in lupus pathogenesis via regulating the above lupus-related inflammatory cytokines. In addition, we also investigated IL-10, an immunomodulatory cytokine that is highly upregulated in human and murine lupus [42]. We utilized antagomir to inhibit miRNA expression in splenic cells because primary lymphocytes can uptake antagomir efficiently to silence specific target miRNA without using any transfection reagent [39, 40]. After 24hrs of antagomir treatment, the expression of targeted DLK1-Dio3 miRNA reduced 50–80% when compared to scrambled control antagomir treated cells (S3A–S3E Fig). We also showed that while antagomir-379 reduced miR-379 expression (S3D Fig) significantly, it has no effect on miR-127 expression (S3F Fig), suggesting the specificity of antagomirs. As shown in Fig 6, inhibition of specific DLK1-Dio3 miRNA reduced the production of cytokines in LPS activated splenocytes from MRL-*lpr* mice. Inhibition of miR-154 significantly reduced IFNγ (Fig 6A) and IL-6 (Fig 6C). Inhibition of miR-300 significantly reduced the production of IFNγ (Fig 6A), IL-1β (Fig 6B), and IL-6 (Fig 6C). Inhibiting miR-300 also reduced the production of IL-10 (Fig 6D, *p* = 0.06) and TNFα (Fig 6E, *p* = 0.067), but the inhibitory effect is not statistically significant. Further, we observed a significant reduction of IFNγ, IL-1β, IL-6, and IL-10 in antagomir-379 treated cells (Fig 6A–6D). It is noteworthy that inhibition of miR-127 had only minor effect on IL-10 (Fig 6D) and that that inhibition of miR-411 had no obvious effect on the production of the above cytokines. Together, our data indicated that DLK1-Dio3 miRNAs might play a role in the regulation of different lupus-related cytokines.

## Discussion

Epigenetic factors including miRNAs and DNA methylation are increasingly recognized as vital contributors to lupus [5, 6]. In this study, we reported that a large cluster of miRNAs from the genomic imprinted DLK1-Dio3 domain is significantly upregulated in splenic cells from MRL-*lpr* lupus mice when compared to control MRL mice, and that this upregulation is associated with DNA hypomethylation in lupus cells. Moreover, we demonstrated that DLK1-Dio3 miRNAs play a role in regulation of inflammation in lupus by regulating the production of lupus-related cytokines. To our knowledge, this is the first report of DNA methylation regulation of genomic imprinted miRNAs in lupus and the potential role of DLK1-Dio3 miRNA in the regulation of lupus-related cytokines. Together, this study provides new perspective in understanding the interaction between two critical epigenetic factors in lupus etiology.

Previous studies have extensively focused on the involvement of CD4^+^ T cell DNA hypomethylation in lupus since demethylated CD4^+^ T cells, but not CD8^+^ T cells, become autoreactive and are able to induce lupus-like disease in mice [43]. There is limited investigation with regard to the changes of global DNA methylation levels in other immune cell types in lupus. In this study, we found that the global DNA methylation levels are decreased not only in lupus CD4^+^ T cells, but also in purified lupus CD19^+^ B cells, as well as in splenic CD4^-^CD19^-^ cells (Fig 2). Concomitantly, DLK1-Dio3 miRNA are increased in all above cell subsets in MRL-*lpr* mice (Fig 1). It is also noticeable that different DLK1-Dio3 miRNAs are upregulated at varied levels in a specific cell type. For example, miR-154 was upregulated over 100 fold, and miR-300 was upregulated only about 4 fold in purified MRL-*lpr* splenic CD4^+^ T cells (Fig 1B). This suggests that the diverse miRNAs on the DLK1-Dio3 miRNA cluster are differentially regulated in a specific immune cell subset. The dysregulation of DLK1-Dio3 miRNAs was also evident in B6-*lpr* and NZB/W_F1_ lupus mice (such as miR-127 and miR-379) [28, 44], and in human lupus patients (such as miR-134, miR-379, and miR-433)[21, 29]. This implies a potential important contribution of DLK1-Dio3 miRNAs to both murine and human lupus, an aspect not known yet.

DNA demethylation treatment of splenic cells from control MRL mice with 5-aza-CdR significantly promoted the expression of genomic imprinted DLK1-Dio3 miRNAs (Fig 3), which strongly suggested that the upregulation of DLK1-Dio3 miRNAs in splenic cells from MRL-*lpr* mice is associated with global DNA hypomethylation in MRL-*lpr* splenic cells. The 5-aza-CdR treatment had no obvious effect on DLK1-Dio3 miRNAs in splenic cells from MRL-*lpr* mice (Fig 4). It is likely that in MRL-*lpr* mice, DLK1-Dio3 domain is intrinsically hypomethylated and therefore is refractory to further DNA demethylation treatment. 5-aza-CdR treatment promoted DLK1-Dio3 miRNAs in splenocytes and also in diverse immune cell subsets (Figs 3 and 5). However, the upregulation of DLK1-Dio3 miRNAs was more profound in CD4^-^CD19^-^ cells when compared to CD4^+^ T cells and CD19^+^ B cells (Fig 5). This suggests DLK1-Dio3 miRNA expression is more sensitive to DNA methylation regulation in CD8^+^ or myeloid cells, which are enriched in splenic CD4^-^CD19^-^ cell population. It will be of particular interest to investigate DNA methylation and miRNAs dysregulation in CD8^+^ or myeloid cells, which are currently neglected with regard to the contribution of their epigenetic changes to lupus. Since DNA demethylation agent 5-azacytidine (5-aza-C) and its deoxy derivative 5-aza-CdR are effective in dividing cells by inhibiting DNA methylation of newly synthesized DNA [43], it is not surprising that 5-aza-CdR treatment increased DLK1-Dio3 miRNA in Con A activated splenocytes (Con A), but not in unstimulated cells (medium, Fig 3A). However, for unknown reason, 5-aza-CdR treatment increased miR-154, miR-127, and miR-379 expression even in inactivated CD4^+^ T cells from MRL mice (medium, Fig 3B), and Con A activation did not further promote the effect of 5-aza-CdR on DLK1-Do3 miRNA expression in CD4^+^ T cells.

DLK1-Dio3 miRNA are differentially expressed in various human pathological conditions and have been implicated in the pathogenesis of various human diseases, especially cancers[29]. The dysregulated expression of selected DLK1-Dio3 miRNAs such as miR-134, miR-433, miR-494, and miR-379 has also been noticed in human lupus [21, 29]. In a previous purely computational prediction analysis, DLK1-Dio3 miRNAs were predicted to target numerous lupus susceptibility genes [45]. However, there is so far no direct experimental data to support the role of this imprinted miRNA cluster in the development of lupus and other autoimmune disorders. We reported here that deliberate inhibition of selected DLK1-Dio3 miRNAs such as miR-154, miR-379, and miR-300 significantly reduced the production of lupus-associated cytokines IFNγ, IL-1β, IL-6, and/or IL-10 (Fig 6). Conceivably, upregulated DLK1-Dio3 miRNAs such as miR-154, miR-379, and miR-300 might accelerate lupus by promoting the production of lupus-related cytokines. Targeting these miRNAs may have potential therapeutic applications in ameliorating lupus manifestation by reducing lupus-related inflammatory cytokines. miR-154, miR-379, and miR-300 have been shown to be decreased in different types of cancer cells, and they function as tumor suppressors by targeting TLR2, Cyclin B1, and Twist, respectively [46–48]. Further studies are needed to determine the target genes of miR-154, miR-379, and miR-300 in immune cells in a lupus setting, an aspect not yet known. This is critical for a better understanding of the molecular mechanism by which DLK1-Dio3 miRNA regulate inflammation.

The imprinting expression of DLK1-Dio3 genes is primarily regulated by the germline-derived intergenic DMR (IG-DMR), which functions as the imprinting control region (ICR) for DLK1-Dio3 locus [30, 49]. Target deletion of IG-DMR in maternally, but not paternally, inherited chromosome leads to bidirectional loss of imprinting of DLK1-Dio3 genes[49]. This suggests the importance of hypomethylated IG-DMR at the maternal chromosome in the repression of paternally expressed protein-coding genes and activation of maternally expressed noncoding RNAs and miRNAs [30, 49]. The secondary, somatic Gtl2-DMR (also called MEG3-DMR in humans) is also hypomethylated at the maternal allele and critically involved in the imprinting of DLK1-Dio3 genes [50, 51]. The loss of genomic imprinting (LOI) expression of DLK1-Dio3 miRNAs in acute promyelocytic leukemia (APL) and type 2 diabetes mellitus (T2DM) has been associated with altered DNA methylation at Gtl2 (MEG3)-DMR region [52, 53]. In addition, a recent study reported a new maternally methylated DMR named CGI-2-DMR, which acquires differential methylation pattern during embryonic development [54]. However, the role of CGI-2 DMR in the regulation of imprinting DLK1-Dio3 gene expression has not been addressed in the report. While our data revealed a positive correlation between DNA hypomethylation and upregulation of DLK1-Dio3 miRNA in MRL-*lpr* mice, the direct link between the DLK1-Dio3 miRNA expression and the differential DNA methylation of DLK1-Dio3 domain is not addressed in the current study. A quick survey of the IG-DMR and Gtl2-DMR with combined bisulfite restriction analysis (COBRA) did not reveal any differentially methylated sites in splenocytes of MRL and MRL-*lpr* mice (Data not shown). In addition to the regulation by DMRs, the expression of a specific DLK1-Dio3 miRNA is also regulated by the CpG enriched regions that are embedded in, or close to the miRNA coding sequences [33]. Therefore, a full, high throughput methylation profiling study is needed to identify the differentially methylated sites at specific DLK1-Dio3 domains such as DMRs and/or CpG enrich regions located at the two major miRNA coding region, *asRTL1* and *Mirg* between MRL and MRL-*lpr* mice, which lead to the LOI and upregulation of DLK1-Dio3 miRNAs directly in lupus. Moreover, it is of particular interest to investigate whether some known lupus-related environmental factors such as endocrine disruptor chemicals and lupus-inducing drugs will affect DNA methylation at DLK1-Dio3 domain, especially during critical and vulnerable developmental stages, to induce abnormal DLK1-Dio3 miRNAs expression and autoimmunity.

While the current study focused on the DNA methylation regulation of genomic imprinted DLK1-Dio3 miRNAs in lupus, it is noteworthy that DNA methylation may interact with histone acetylation to regulate the imprinting of DLK1-Dio3 locus[55]. It is necessary to investigate the potential involvement of histone modification alteration in the LOI and dysregulation of DLK1-Dio3 miRNAs in lupus in future study. Further, different mechanism other than LOI may be also involved in the upregulation of DLK1-Dio3 miRNAs in lupus. Together, our novel data provides a connection among DNA methylation, miRNA, and genomic imprinting, which may facilitate a better understanding of lupus etiology.

## Supporting Information

S1 Fig Test the effect of 5-aza-CdR treatment on splenic cell viability. The splenocytes and purified CD4^+^ T cells from MRL mice were treated with vehicle solution (DMSO) or 5-aza-CdR (AZA, 2μM or 5μM), with (Con A) or without (medium) Con A (5μg/ml) activation for 72 hrs. After treatment, aliquot of the cells were stained with propidium iodide and then subjected to Flow cytometric analysis. The graph shows the percentages of viable cells after 72hrs of treatment in each treatment condition (means± SEM, n = 5 each). Paired student *t* tests were performed (Vehicle *vs* AZA); *, *p* < 0.05; **, *p* < 0.01; and ***, *p* < 0.001.
(TIF)

S2 Fig DLK1-Dio3 miRNA is differentially expressed in diverse splenic cell subsets. The DLK1-Dio3 miRNA expression levels in splenocytes, purified CD4^+^ T cells, CD19^+^ B cells, and splenic CD4^-^CD19^-^ cells from MRL (A) and MRL-*lpr* (B) mice were quantified by Taqman miRNA assays. The expression level of a specific DLK1-Dio3 miRNA in splenic CD4^+^ T, CD19^+^ B, and CD4^-^CD19^-^ cells was referred to the level in splenocytes. The graphs show means ± SEM (n = 3). To assess the statistical significance of the expression levels of a specific miRNA between different splenic cell subsets in the same mouse strain, One-way ANOVA analysis was performed with JMP Pro software (version 11, from SAS Institute Inc, Cary, NC, USA). All pairs, Tukey-Kramer HSD (honestly significant difference) tests were performed to compare the means of each miRNA in splenocytes and different cell subsets. A letter-coded report was generated by the software to depict the statistical significance of differences among the means of multiple groups. The means that are not sharing an alphabetic letter (for example, *a vs b vs c*) are significantly different. The means that are sharing an alphabetic letter (for example, *a vs a; b vs b; a vs a/b; b vs a/b*) are not significantly different.
(TIF)

S3 Fig DLK1-Dio3 miRNA antagomirs suppress the respective specific miRNA efficiently. The splenocytes from MRL-*lpr* mice were treated with either scrambled control or specific DLK1-Dio3 miRNA antagomirs such as antagomir-127 (A), antagomir-154 (B), antagomir-300 (C), antagomir-379 (D), and antagomir-411 (E) for 24hrs, and then collected to analyze miRNA expression. The expression level of miR-379 was analyzed in antagomir-127 treated cells to show the specificity of antagomir (F). The graphs show means ± SEM (n = 2).
(TIF)

S1 Table Scrambled control and specific DLK1-Dio3 miRNA antagomirs sequences. (TIF)
