# Supplementary figures and images for: The Upregulation of Genomic Imprinted DLK1-Dio3 miRNAs in Murine Lupus Is Associated with Global DNA Hypomethylation
Source: PLoS One. 2016 Apr 12;11(4):e0153509. doi: 10.1371/journal.pone.0153509 (PMC4829153; doi:10.1371/journal.pone.0153509)

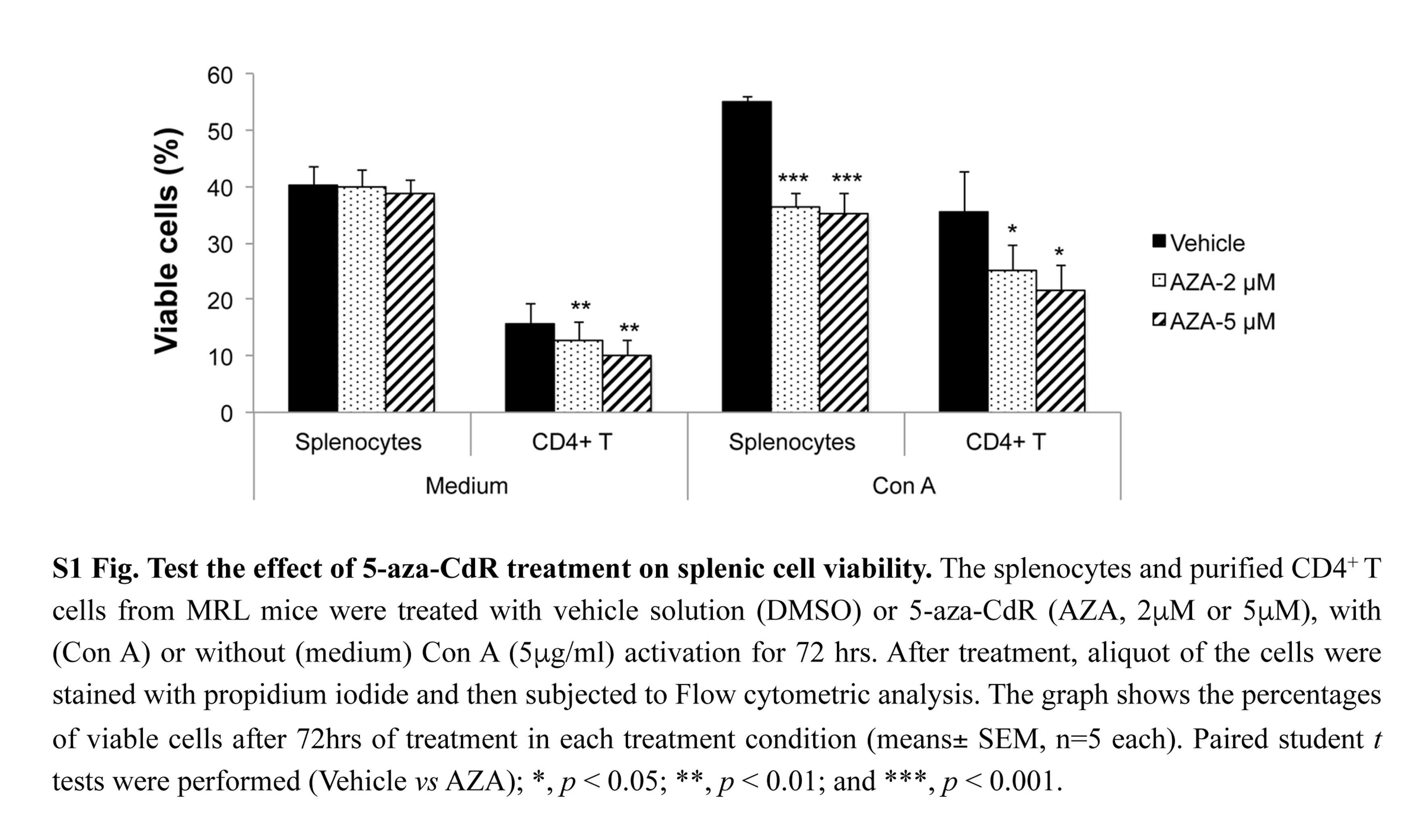

Supplement: S1 Fig — The splenocytes and purified CD4+ T cells from MRL mice were treated with vehicle solution (DMSO) or 5-aza-CdR (AZA, 2μM or 5μM), with (Con A) or without (medium) Con A (5μg/ml) activation for 72 hrs. After treatment, aliquot of the cells were stained with propidium iodide and then subjected to Flow cytometric analysis. The graph shows the percentages of viable cells after 72hrs of treatment in each treatment condition (means± SEM, n = 5 each). Paired student t tests were performed (Vehicle vs AZA); *, p < 0.05; **, p < 0.01; and ***, p < 0.001. (TIF) [file pone.0153509.s001.tif]

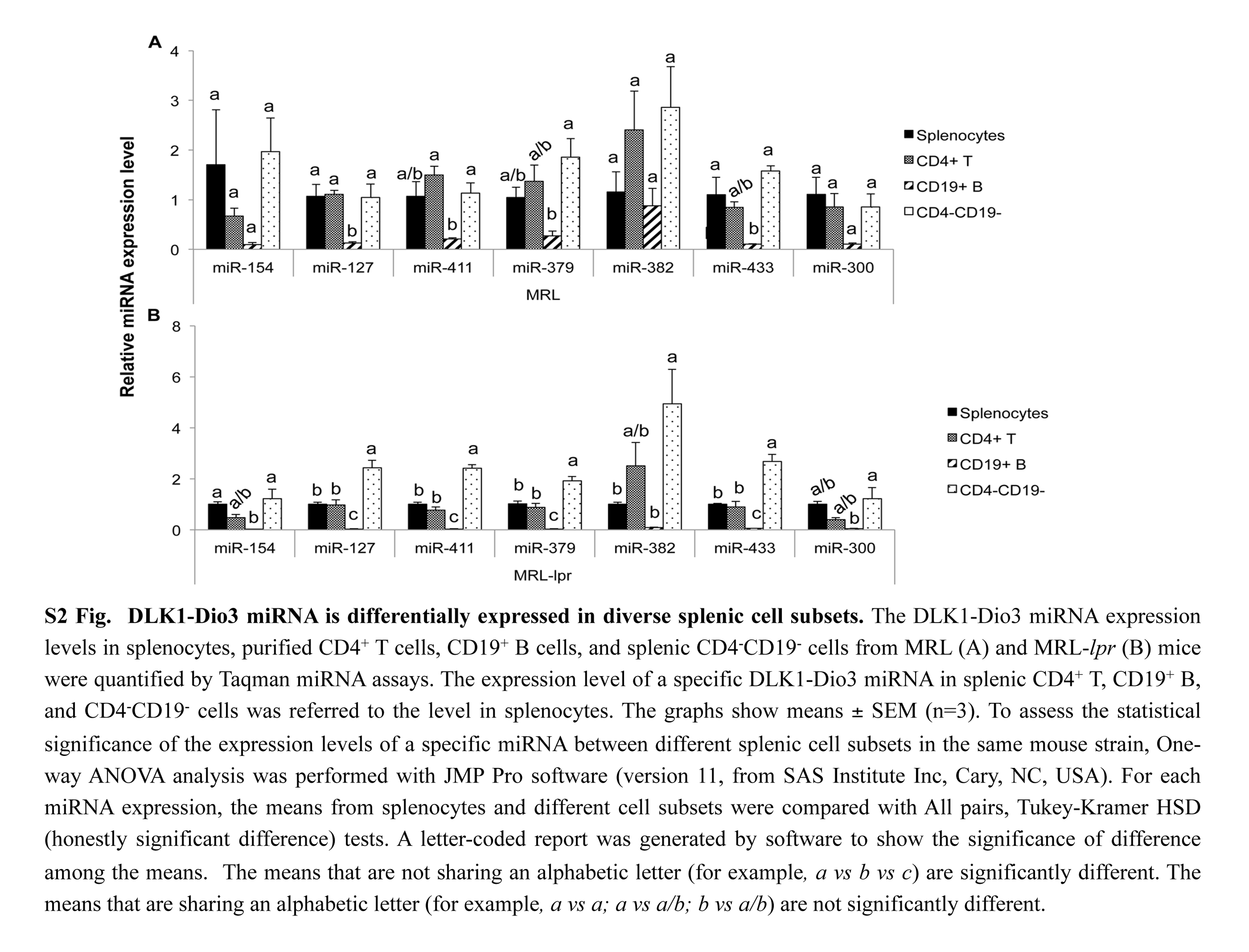

Supplement: S2 Fig — The DLK1-Dio3 miRNA expression levels in splenocytes, purified CD4+ T cells, CD19+ B cells, and splenic CD4-CD19- cells from MRL (A) and MRL-lpr (B) mice were quantified by Taqman miRNA assays. The expression level of a specific DLK1-Dio3 miRNA in splenic CD4+ T, CD19+ B, and CD4-CD19- cells was referred to the level in splenocytes. The graphs show means ± SEM (n = 3). To assess the statistical significance of the expression levels of a specific miRNA between different splenic cell subsets in the same mouse strain, One-way ANOVA analysis was performed with JMP Pro software (version 11, from SAS Institute Inc, Cary, NC, USA). All pairs, Tukey-Kramer HSD (honestly significant difference) tests were performed to compare the means of each miRNA in splenocytes and different cell subsets. A letter-coded report was generated by the software to depict the statistical significance of differences among the means of multiple groups. The means that are not sharing an alphabetic letter (for example, a vs b vs c) are significantly different. The means that are sharing an alphabetic letter (for example, a vs a; b vs b; a vs a/b; b vs a/b) are not significantly different. (TIF) [file pone.0153509.s002.tif]

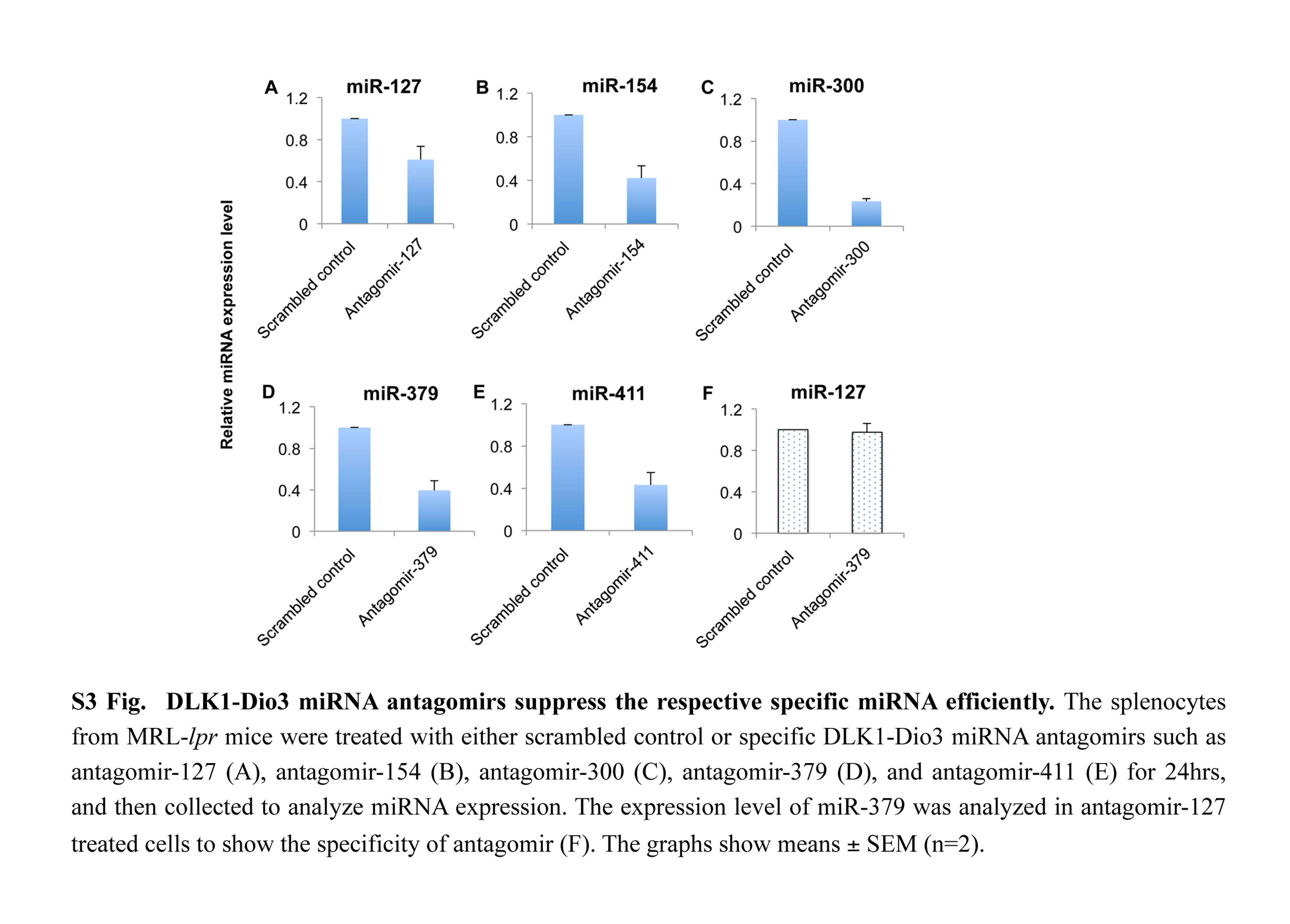

Supplement: S3 Fig — The splenocytes from MRL-lpr mice were treated with either scrambled control or specific DLK1-Dio3 miRNA antagomirs such as antagomir-127 (A), antagomir-154 (B), antagomir-300 (C), antagomir-379 (D), and antagomir-411 (E) for 24hrs, and then collected to analyze miRNA expression. The expression level of miR-379 was analyzed in antagomir-127 treated cells to show the specificity of antagomir (F). The graphs show means ± SEM (n = 2). (TIF) [file pone.0153509.s003.tif]

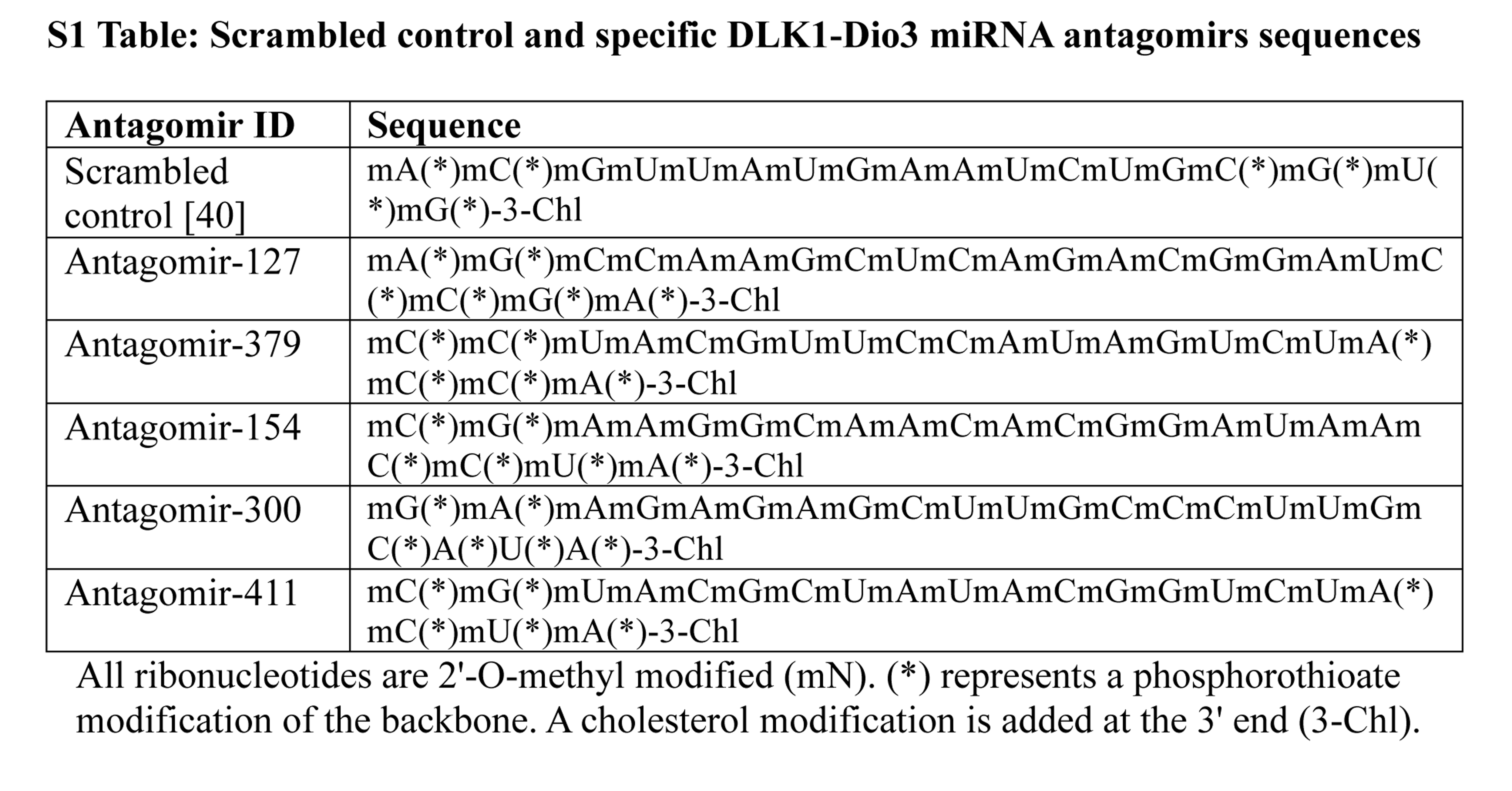

Supplement: S1 Table — (TIF) [file pone.0153509.s004.tif]
